# Distal Transradial Access Optimization: A Prospective Trial of Ultrasound-Guided Radial Artery Characterization for the Anatomical Snuffbox

**DOI:** 10.3390/diagnostics14182081

**Published:** 2024-09-20

**Authors:** Łukasz Koziński, Zbigniew Orzałkiewicz, Paweł Zagożdżon, Alicja Dąbrowska-Kugacka

**Affiliations:** 1 Department of Cardiology, Chojnice Specialist Hospital, Lesna 10, 89-600 Chojnice, Poland; zorzel11@wp.pl; 2 Department of Hygiene and Epidemiology, Medical University of Gdansk, Debinki 7, 80-211 Gdansk, Poland; pzagoz@gumed.edu.pl; 3 Department of Cardiology and Electrotherapy, Medical University of Gdansk, Smoluchowskiego 17, 80-214 Gdansk, Poland; alicja.dabrowska-kugacka@gumed.edu.pl

**Keywords:** distal transradial access, snuffbox approach, distal radial artery, radial artery, vascular ultrasound, radial ultrasound, radial artery pulse

## Abstract

**Background/Objectives:** The distal transradial approach (dTRA) is increasingly used in interventional cardiology. Doppler Ultrasound (DUS) effectively assesses radial artery (RA) characteristics. This study aims to identify specific RA DUS characteristics in patients undergoing coronary procedures via dTRA. **Methods**: Participants from the ANTARES trial who completed the intervention per-protocol and retained RA patency were included. DUS was performed at baseline, 1 day, and 60 days post-procedure. **Results**: Among 400 participants, 348 had either dTRA (n = 169) or conventional transradial access (cTRA) (n = 179). Distal RA lumen diameter was 12% smaller than that of the proximal RA (*p* < 0.001). Men had a 14% larger distal RA diameter than women (2.33 ± 0.31 mm vs. 2.04 ± 0.27 mm, *p* < 0.0001), similar to the proximal RA relationship. Peak flow velocities were similar between the sexes. Univariate linear regression showed that height, weight, body mass index, and body surface area (BSA) predicted arterial size, with BSA remaining significant in multivariate analysis (beta coefficient 0.62; confidence interval 0.49–0.75; *p* < 0.0001). Distal RA diameter correlated positively with palpable pulse at the snuffbox and wrist. The dTRA resulted in an immediate 14% and 11% increase in distal and proximal RA diameter, respectively (both *p* < 0.05). Sixty days after dTRA, the distal RA remained slightly dilated (*p* < 0.05), while the proximal RA returned to baseline. **Conclusions**: Distal RA diameter is significantly associated with sex, measuring smaller than the forearm segment. A strong palpable pulse correlates with larger distal RA size. The dTRA induces RA lumen expansion. A thorough understanding of distal RA anatomy is essential for optimizing patient selection and refining techniques for transradial procedures.

## 1. Introduction

Coronary angiography (CAG) and percutaneous coronary intervention (PCI) are at the forefront of coronary artery disease management, serving as the cornerstone diagnostic and therapeutic modalities in contemporary cardiology. There has been a significant paradigm shift in vascular access methodologies for CAG and PCI, primarily directed towards reducing patient discomfort and diminishing procedural complications. Over the past three decades, conventional transradial access (cTRA) has been extensively researched and solidified its position as a preferred technique by virtue of its substantial benefits; these include a marked decrease in major hemorrhagic events at the access site and improved patient comfort, alongside notable cost-saving implications in comparison to the transfemoral route [1,2,3,4].

Currently, both European and American societies recommend the standard use of vascular access via the radial artery (RA), particularly in patients with acute coronary syndromes, in whom this practice contributes to reducing mortality rates [3,4].

The distal transradial approach (dTRA), targeting the distal segment of the RA within the anatomical snuffbox, has been acknowledged for potentially mitigating the incidence of RA occlusion in comparison to its conventional counterpart [5,6]. Acclaimed for expediting hemostasis and reducing vascular complications, dTRA is accruing international acceptance as a viable alternative for coronary angiography and interventions. Nevertheless, its adoption is tempered by the technical intricacies of arterial cannulation. Empirical evidence from various clinical trials indicates an inclination towards increased attempts at arterial access, prolonged sheath insertion time, and a consequent escalation in crossovers to alternate access sites with dTRA [5,7,8]. These observed trends are conjectured to be consequent to the specific anatomical nuances of the distal RA, warranting further exploration and adaptation of technique to leverage the full potential of dTRA [9].

Ultrasonographic evaluation plays a pivotal role in delineating the anatomy of the radial artery and in determining its aptness for cTRA [10,11]. Doppler Ultrasound (DUS) represents a straightforward, accessible, cost-efficient, repeatable, and non-invasive modality for the analysis of the vascular architecture. Given these advantages, DUS proves instrumental in examining the distal RA. Pertinent research underscores the utility of sonographic appraisal for ascertaining the viability and success probabilities of dTRA [12,13,14]. Sonographic imaging enables precise measurements of arterial diameter, assessment of tortuosity, identification of dual arterial supply, along with other morphological parameters, thereby facilitating the selection of suitable candidates for this emergent access strategy. The critical data garnered from such evaluations are instrumental in optimizing the strategies for coronary interventions.

The objective of this study was to assess the DUS characteristics of the distal RA in the Caucasian population both pre- and post-procedure. Specifically, this study sought to distinguish gender-specific attributes, correlate the palpability of radial pulse strength with vessel dimensions, and identify additional anatomical and clinical determinants impacting radial artery caliber.

## 2. Materials and Methods

### 2.1. Study Design and Population

This study’s inclusion criteria targeted Caucasian individuals aged 18 years or older who were eligible for CAG/PCI in line with existing medical guidelines and who provided informed consent for trial participation. From an initial cohort of 465 consecutive patient evaluations, 400 patients met the criteria for inclusion, forming the study cohort of the previously documented ANTARES trial: a single-center, randomized (1:1 allocation; dTRA vs. cTRA), and prospective study [15]. Exclusion criteria were applied rigorously, ruling out individuals with ST-elevation myocardial infarction, incidents of sudden cardiac arrest, hemodynamic instability, those in advanced stages of chronic kidney disease (stages 4–5), patients with existing occlusions in forearm arteries, prior unsuccessful attempts of ipsilateral transradial access, radial arteries deemed unsuitable in diameter, and cases where DUS was infeasible. Part of the analysis encompassed only participants who adhered to the treatment-per-protocol follow-up, without recourse to access crossovers, and who maintained RA patency post-procedure.

All participants provided written informed consent before enrolling in this study. The study protocol adhered to the standards outlined in the Declaration of Helsinki and received approval from the Bioethical Commission at the Regional Medical Chamber (KB–25/18), NCT05982366.

### 2.2. Ultrasonography

DUS examinations were conducted by an invasive cardiology operator with prior experience in forearm artery scans for 200 patients. A commercially available Vivid 7 sonographic machine (General Electric Healthcare, Milwaukee, WI, USA) equipped with the linear-array transducer ultrasound probe, operating at an average frequency range of 8.0–10 MHz, was optimized for the detailed study of arterial structures in the upper extremities. These examinations were carried out in a controlled environment, maintaining the standard room temperature range of 20 to 22 degrees Celsius, within an adequately tranquil and dimly illuminated laboratory setting, ensuring that the patients had an adequate relaxation period beforehand.

This study included vascular evaluations at three specific time points: within 24 h before the intervention, one day post-procedure, and at the sixty-day mark following the procedure. Initial participant assessment entailed an eligibility screening in line with the trial’s criteria. This preliminary evaluation ascertained the patency of the radial artery at both conventional and distal access points, in addition to confirming the ulnar artery’s patency. To ensure the vessels were suitable for puncture, we set a criterion that distal RA lumen dimension should measure at least 1.8 mm in the radial fossa, based on our prior successful cannulation experiences preceding this study’s enrollment. 

The measurements of the forearm arteries were taken in a seated patient, with the forearm in supination, fully supported, and bent at a 90-degree angle at the elbow joint. Pre-procedural examinations involved assessing both the RA and ulnar artery, whereas subsequent follow-up assessments focused solely on the RA. The measuring point for the distal RA was located within the anatomical snuffbox (over the scaphoid and trapezium bones in ultrasound), while for the proximal RA, it was approximately 2 cm proximal to the styloid process of the radius bone. The ulnar artery was measured 2 cm proximal to the wrist. 

**Figure 1:**
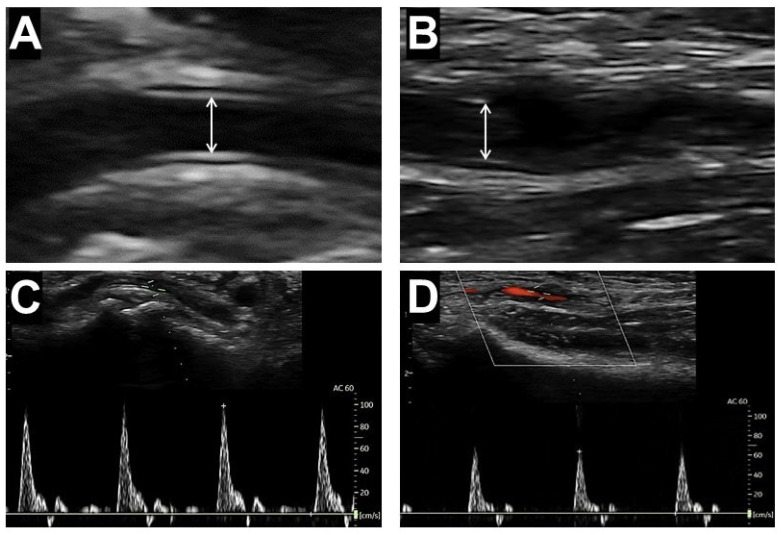
Ultrasound measurement of lumen diameters and blood flow velocities in the distal radial artery (**A**,**C**) and forearm radial artery (**B**,**D**). Arrows indicate the luminal diameter of the artery, measured as the distance between the intimal layers of the intima-media complex.

The vessel luminal caliber, defined as the largest distance between the internal hyperechogenic lines of the intima–media complex of the arterial wall in the antero-posterior dimension, was measured during the diastolic phase determined using echo-tracking B-mode ultrasound scanning (Figure 1A,B). The blood peak flow velocities (PFVs) of the arteries were measured at the locations of vessel diameter measurements, using the pulsed-wave Doppler scanner set parallel to the blood flow based on color-Doppler imaging (Figure 1C,D). The values of size and PFV are the averaged results from three consecutive measurements. 

### 2.3. Radial Artery Access

A detailed description of the vascular access procedure was provided in a previous publication [15]. Local anesthesia preceded vessel puncture, and ultrasound was not utilized for guided access. Radial artery puncture, usually at a 30–45° angle using a 22-gauge needle and Seldinger technique, followed with verapamil injection for vasodilation. Heparin was administered during the procedure. Ten-centimeter-long hydrophilic transradial kits were employed, including the 5-Fr/6-Fr Radifocus^®^ Introducer II and 6-Fr Glidesheath Slender^®^ Introducer (Terumo Corp., Tokyo, Japan). The selection of the sheath size was at the discretion of the operator. At the end of the procedure, the sheath was removed, and a compression dressing was applied, maintained for 4 h following coronary angiography and 5 h following percutaneous coronary intervention.

### 2.4. Follow-Up and Study Endpoints

This study assessed the diameter and blood flow velocity of the distal RA, including gender-related differences and comparisons with the proximal RA. It also evaluated the relationship between distal RA diameter and manually assessed pulse strength, explored correlations between distal RA size and anatomical or clinical factors, and monitored changes in size and blood flow following the procedure. The first DUS follow-up was conducted 24 h post-procedure, with another follow-up at 60 days.

### 2.5. Statistical Analysis

Data on anatomical, demographic, clinical, angiographic, procedural, and ultrasonographic parameters were prospectively recorded in a computerized database. The Shapiro–Wilk test was utilized to assess their distribution. Continuous variables were presented as mean ± standard deviation (SD) and compared using an unpaired *t*-test or Mann–Whitney U test, where appropriate. For non-normally distributed data, the median with the interquartile range (IQR) was presented. Categorical variables were displayed as absolute or relative frequencies and compared using chi-square analyses or Fisher’s exact test, as appropriate to the cell frequencies. 

The linear regression method was applied to identify variables that independently predicted radial artery diameter. Predictors with a univariate *p*-value ≤ 0.2 were included in the multivariate linear regression model. The comparison between 3 time slots of DUS was performed with repeated measures ANOVA analysis and the post hoc Tukey test. 

A *p*-value < 0.05 was considered statistically significant. STATA software (version 17, StatCorp) was used to calculate statistics.

## 3. Results

### 3.1. Patients and Procedural Characteristics

**Figure 2:**
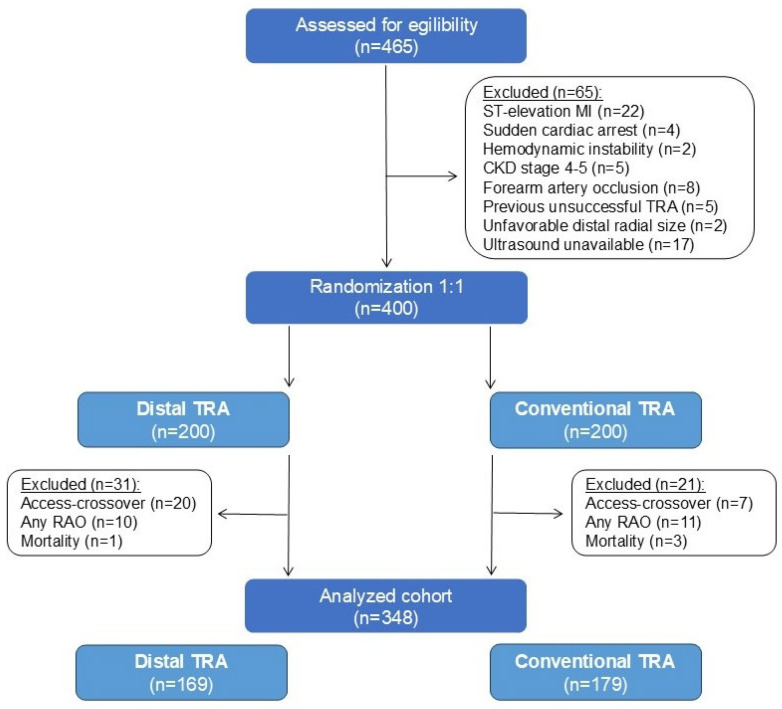
Study flow chart. CKD = chronic kidney disease; MI = myocardial infarction; RAO = radial artery occlusion; TRA = transradial approach.

The patient study flow is depicted in Figure 2. Over a span of four months, 400 patients were recruited, among whom the lumen diameter of the RA was assessed at baseline and evaluated based on gender. Subsequently, after meeting further qualification criteria, i.e., maintaining artery patency during follow-up according to intervention per-protocol (without access crossover), further analyses were conducted. A total of 348 patients were then assigned to either the dTRA (n = 169) or cTRA (n = 179) groups. None of the patients were lost during the follow-up period of up to 60 days. 

Baseline clinical and procedural characteristics for all patients are detailed in Table 1 and Table 2. For the overall sample, the average age was 66.6 ± 10 years, with 37% being female. Nearly 80% of patients presented with hypertension, 37% with diabetes, and 44% were classified as obese (BMI > 30), while 29% had undergone previous PCI. The majority of patients were scheduled for coronary procedures due to chronic coronary syndrome (58%), with non-ST-elevation acute coronary syndrome accounting for over 30% of cases. Over 24% of patients had a history of ipsilateral TRA. Nearly 62% of vascular accesses were right-sided. The dTRA and cTRA groups did not differ in terms of the severity of coronary artery disease, the frequency of PCI (37.9% vs. 39.7%), fluoroscopy time (median (M) (interquartile range (IQR)) 3.76 (1.90–8.31) vs. 4.07 (2.00–7.74) minutes), or total procedure time (M (IQR) 17.0 (10.3–34.0) vs. 16.5 (9.7–30.5) minutes). There were no differences in baseline clinical characteristics and most procedural data between the dTRA and cTRA cohorts. 

The sole procedural parameters that differed between the cohorts were an extended access performance time (M (IQR) 115 (80–230) vs. 80 (55–120) seconds; *p* < 0.001) and a preference for smaller vascular sheath sizes (5Fr sheaths: 51.5% vs. 31.8%; *p* < 0.001 and 6Fr sheaths: 43.8% vs. 68.2%; *p* < 0.001) in the dTRA group.

### 3.2. Baseline Ultrasound RA Characteristics

**Figure 3:**
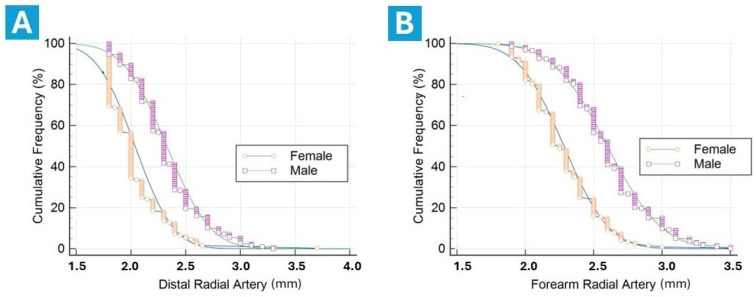
Gender-related cumulative frequencies of lumen diameters in the distal radial artery (**A**) and forearm radial artery (**B**).

Baseline artery ultrasound measurements are illustrated in Table 3. The sizes of forearm arteries, namely the RA at its distal part in the snuffbox area and at its proximal part in the forearm, as well as the ulnar artery, were significantly larger among males (Figure 3). The lumen diameter of the distal RA in males was over 14% larger than in females (2.33 ± 0.31 mm vs. 2.04 ± 0.27 mm; *p* < 0.0001). An identical difference (14%) between genders was observed when comparing the proximal RA size. The mean dimension of the distal RA was significantly smaller than the proximal RA by over 12% (2.21 ± 0.33 mm vs. 2.47 ± 0.33 mm; *p* < 0.0001), and this relationship was consistent regardless of gender. 

There were no differences in blood PFV in the RA between genders, but statistically significantly lower PFVs were observed in the distal compared to proximal RA (0.58 ± 0.16 m/s vs. 0.61 ± 0.15 m/s; *p* < 0.05).

### 3.3. Palpable Pulse Strength in Relation to RA Size

**Figure 4:**
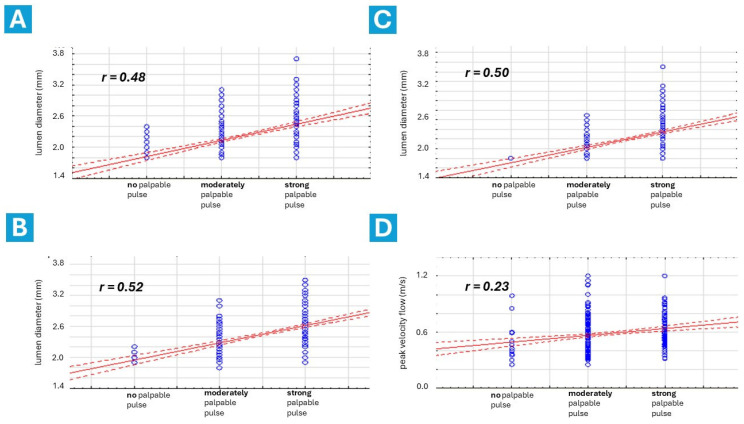
Correlation between radial artery size, blood peak velocity flow, and strength of palpable pulse at the snuffbox and forearm. Correlation between distal radial artery size and palpable pulse strength at the snuffbox (**A**), forearm radial artery size and palpable pulse strength at the wrist (**B**), distal radial artery size and palpable pulse strength at the wrist (**C**), distal radial artery size and blood peak velocity flow at the snuffbox (**D**). r = correlation coefficient.

Data regarding these issues are presented in Table 1 and Figure 4. The palpatory assessment of RA pulse was categorized into three levels: no pulse, moderately palpable pulse, and strong palpable pulse. No differences were found in the pulse assessment of the RA between the dTRA and cTRA groups; however, a stronger pulse was observed more frequently in the proximal RA than in the distal RA (*p* < 0.05). A positive correlation was found between baseline distal RA size and manually assessed palpable pulse at the snuffbox (r = 0.48) and forearm (r = 0.50). A weak correlation was found between distal RA size and PFV (r = 0.23).

### 3.4. Factors Affecting RA Size

Table 4 shows univariate predictors of RA size. In the simple linear regression analysis, height (beta coefficient (β) 1.19, 95% CI (confidence interval) 0.874–1.514, *p* < 0.0001), weight (β 0.007, 95% CI 0.006–0.009, *p* < 0.0001), body mass index (BMI) (β 0.013, 95% CI 0.008–0.020, *p* < 0.0001), and body surface area (BSA) (β 0.62, 95% CI 0.490–0.757, *p* < 0.0001) were predictors of distal RA dimension. The same factors were predictors of proximal RA size. The univariate analysis suggests that larger height was associated with a bigger RA lumen diameter, while larger weight, BMI, and BSA corresponded to a smaller diameter. After accounting for the interplay of various factors, neither hypertension nor diabetes, nor any other factors examined, showed a significant association with RA size. In the stepwise multiple regression model, only BSA emerges as an independent factor influencing the size of the RA (β 0.62; CI 0.49–0.75; *p* < 0.0001).

### 3.5. Post-Procedural Ultrasound RA Characteristics

**Figure 5:**
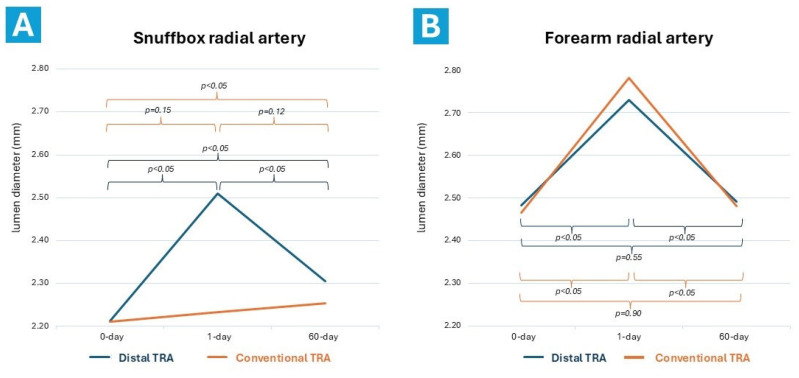
Differences in radial artery lumen size changes at the snuffbox (**A**) and forearm (**B**) at baseline, 1 day, and 60 days after distal and conventional transradial approaches. TRA = transradial approach; mm = millimeter.

**Figure 6:**
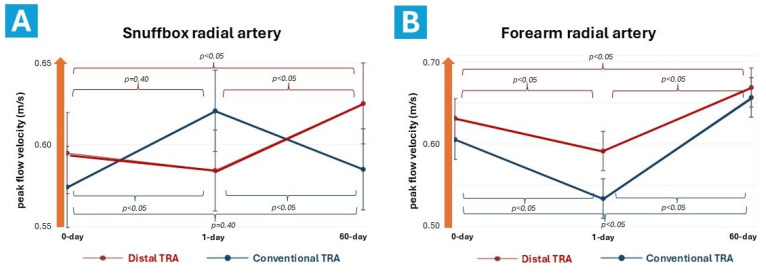
Differences in radial artery peak flow velocity dynamics at the snuffbox (**A**) and forearm (**B**) at baseline, 1 day, and 60 days after distal and conventional transradial approaches. TRA = transradial approach; m/s = meter per second.

Data on pre- and post-procedural radial artery dimension and blood velocity flow are shown in Table 5 and Figure 5 and Figure 6. Distal TRA resulted in a statistically significant increase in the diameter of the distal RA by over 14% after 24 h post-procedure (from 2.24 ± 0.31 mm to 2.55 ± 0.30 mm; *p* < 0.001). The enlargement effect diminished after 60 days post-procedure, but distal RA size still remained larger than at baseline (from 2.24 ± 0.31 mm to 2.34 ± 0.32 mm; *p* < 0.05). Distal TRA also resulted in a significant increase in the diameter of the proximal RA (from 2.52 ± 0.33 to 2.75 ± 0.32 mm; *p* < 0.001), though this increase was nullified by the 60-day follow-up. One day after the procedure, conventional TRA led to an increase in RA size at the puncture site of over 13%, but had no effect on the dimension of the distal RA. Two months after cTRA, the proximal RA size returned to baseline value, but the distal RA experienced a slight yet significant increase. 

Conventional TRA resulted in a decrease in PFV at the puncture site 24 h post-procedure (*p* < 0.05), while dTRA did not affect blood flow at its puncture site immediately after the procedure (*p* = 0.40). Both approaches led to an increase in PFV at the puncture site 60 days after the procedure compared to baseline values (*p* < 0.05). The dTRA was associated with a post-procedural decrease in PFV in the proximal RA (*p* < 0.05), followed by an increase after 60 days relative to baseline (*p* < 0.05). Only cTRA caused an increase in PFV in the distal RA immediately after the procedure (*p* < 0.05), which returned to baseline value during the longer follow-up period (*p* = 0.40).

## 4. Discussion

Our study is one of the few that evaluated the ultrasound features of the distal RA before and after dTRA, with a follow-up of up to 2 months. This study reveals significant anatomical insights regarding the RA, with implications for transradial access procedures. Our findings demonstrate a clear sex-based difference in RA size, with men exhibiting a 14% larger diameter compared to women. Furthermore, the distal RA was observed to be approximately 12% smaller than the RA segment in the forearm. Importantly, anthropometric parameters, including height, weight, BMI, and BSA, were found to be predictive of distal RA size. Among these, BSA remained a significant predictor in multivariate analysis. Interestingly, distal RA diameter exhibited a transient increase immediately following dTRA, with a subsequent decrease in its dimensions observed within a few weeks. Finally, a positive correlation was observed between the strength of palpable pulse at the RA, assessed at both the distal and forearm locations, and the diameter of the distal RA. This finding underscores the importance of meticulous physical examination in evaluating this access suitability and potentially predicting procedural success. 

Pre-procedural DUS assessment of distal RA diameter is paramount for optimizing patient selection and tailoring instrumentation for dTRA, thereby minimizing the risk of iatrogenic RA injury. As the adoption of the dTRA expands across varying operator experience levels and encompasses increasingly complex patient profiles and interventions, a thorough understanding of the distal RA’s characteristics, including its inherent limitations, becomes essential for ensuring procedural safety and efficacy.

The anatomical snuffbox, delineated by the tendons of the abductor pollicis longus and extensor pollicis brevis laterally and extensor pollicis longus medially, provides a novel access point for the dTRA. This area has a distinct anatomy, with the RA residing close to the surface and relatively free from overlying structures, which substantiates the use of this vascular route [9,16,17]. It not only permits a more superficial and direct puncture but also mitigates the risk of neurovascular damage [12]. The RA in this location, although smaller and potentially more prone to vasospasm or tortuosity, is advantageous due to lower rates of occlusion and faster hemostatic outcomes compared to the traditional forearm access site [6,7,8,9,12,16,17]. 

While the dTRA via the anatomical snuffbox offers numerous advantages, inherent anatomical challenges warrant careful consideration. The smaller caliber and increased tortuosity of the distal RA can complicate puncture attempts. Therefore, clinical decision-making should involve a nuanced assessment balancing these anatomical considerations against the potential benefits of the dTRA, including enhanced patient comfort and a reduction in access-site complications. In this context, DUS guidance emerges as a valuable tool for mitigating procedural risks and enhancing efficiency. By facilitating the visualization of the target artery, accurate determination of arterial diameter for appropriate sheath selection, and overall minimization of access-related difficulties and complications, DUS guidance contributes significantly to the safety and effectiveness of the dTRA [12].

The diameter of the distal RA is a critical anatomical factor influencing the success of the dTRA. Studies have shown that the distal RA tends to be significantly smaller than the proximal RA, with mean diameters ranging from 1.70 to 2.99 mm, and that females have notably smaller diameters than males [18,19,20,21,22,23,24,25,26,27,28,29,30,31]. The substantial disparities in the estimated size of the distal RA, often independent of race, may be related to the different methods used for estimating artery size (DUS or angiography), differences in the location of the measurement point in the anatomical snuffbox area, definitions of vessel dimension (inner diameter from media to media, or outer diameter between lines indicating the adventitia), the resolution of the imaging equipment used, the examiner’s experience, or even the study conditions [27]. The measurements of the distal RA obtained in our study are similar to the results of other European studies, which estimated this dimension at an average of 2.30 ± 0.20 mm and 2.31 ± 0.47 mm [24,31]. Interestingly, the largest average luminal diameter of the distal RA was observed in the Japanese population (2.99 ± 0.60 mm), while the smallest was found in the Chinese population (1.70 ± 0.50 mm) [21,30]. The smaller caliber of the distal RA presents a technical challenge for operators, potentially increasing the risk of arterial spasm or occlusion [12]. The use of larger vascular sheaths and the mismatch between artery and sheath size are recognized as contributing factors to arterial wall injury and RA occlusion [32].

Distal RA diameter is closely associated with its forearm segment size, with a strong positive correlation observed (r = 0.66; *p* < 0.0001) and a size ratio of approximately 80% [18,19,27]. In our study, the ratio of inner diameters was slightly higher, at 89%. Several reports have noted that the diameter of the distal RA is wider than that of the proximal RA, although this finding was observed in only a small percentage of the studied populations [23,27]. A growing body of research has established a correlation between anthropometric and clinical factors and distal RA dimensions. Height, body weight, BMI, and BSA are consistently cited as influential parameters, a finding corroborated by the present study [19,23,25,27]. Furthermore, certain comorbidities, including diabetes and hypertension, have been associated with a reduction in distal RA size [27], which was not the case in our study.

Our study revealed a noteworthy positive correlation between distal RA diameter and the strength of the palpable pulse, both at the anatomical snuffbox (r = 0.48) and the wrist (r = 0.50). This suggests that careful palpation at these locations could potentially provide an initial estimate of distal RA size, guiding the selection of appropriately sized vascular sheaths and other equipment for coronary interventions. However, further investigation is warranted to determine whether palpation alone can reliably replace DUS verification.

Achieving successful distal RA puncture on the first attempt is critical for efficient dTRA. Factors such as psychological stress, local anesthetic infiltration, and repeated puncture attempts can induce arterial spasm, potentially leading to transradial procedure failure. Interestingly, a recent study demonstrated that prophylactic application of a transdermal nitroglycerin patch significantly improved first-pass cannulation success rates in palpation-guided dTRA (59% vs. 24%, *p* = 0.001; OR 4.5, 95% CI 1.9 to 11.0) [33]. The authors attributed this improvement to a reduction in arterial spasm risk and observed an 18% increase in mean distal RA diameter in the nitroglycerin group compared to placebo (3.21 mm vs. 2.71 mm, *p* < 0.001) [33]. As noted in our previous research on this population, arterial vasospasm and tortuosity were the main limitations of dTRA [15]. This was partly attributed to reduced palpable radial artery pulse, potentially caused by local subcutaneous anesthesia or needle punctures, both of which could contribute to spasm [15]. To reduce the frequency of access crossover from dTRA, each method should be considered, including the pre-procedural application of nitroglycerin to the skin surface.

There are considerable uncertainties regarding the behavior of distal RA size after successful dTRA and its potential for reuse as a vascular access site in the future. In our study, we observed an immediate significant average 14% increase in distal RA diameter 24 h post-procedure, from 2.24 ± 0.31 mm to 2.55 ± 0.30 mm (*p* < 0.05). However, over the following two months, a reduction in vessel lumen was observed, though it remained significantly larger (2.34 ± 0.32 mm) than at baseline. For instance, a Japanese trial observed a 9% increase in RA diameter one day after dTRA [20]. Conversely, another study reported no significant difference in distal RA size one month post-procedure [30]. A separate study, utilizing ultra-high-resolution DUS with a 55 MHz transducer, observed a 13% reduction in distal RA luminal diameter 90 days post-dTRA [29]. This study also investigated whether dTRA resulted in less RA intimal hyperplasia compared to cTRA. Interestingly, the researchers found significantly greater intima–media thickness in the proximal RA but no difference in intima–media thickness between the two access points after 90 days. This suggests that while dTRA was initially thought to minimize forearm RA occlusion, the remodeling process, specifically intima–media thickening, might be influenced by factors beyond the puncture site alone, such as the advancement of the sheath into the artery. This finding offers valuable insight into the potential mechanisms underlying the arterial wall response to injury following dTRA. It remains unclear whether radial artery remodeling and potential intimal hyperplasia induced by dTRA will affect the success of future access reuse or influence the selection of sheath size for subsequent procedures. 

This study makes a significant contribution to the field by comprehensively characterizing the ultrasound characteristics of the RA following dTRA. Notably, our analysis encompasses three distinct time points, providing a unique and detailed longitudinal perspective on post-procedural changes in a large patient population. This robust approach allowed for an in-depth assessment of the evolving impact of dTRA on RA morphology and hemodynamics. 

### Study Limitations

While the fact that ultrasonographic measurements were performed by a single, experienced ultrasonographer who also served as the invasive cardiology operator may be considered a limitation, this setup also presents advantages. On one hand, the lack of blinding of the ultrasonographer to group allocation precludes interobserver variability analysis. On the other hand, this same operator and ultrasonographer has a greater incentive to choose the optimal access site, increasing the likelihood of confirming the accuracy of the ultrasound assessment during catheterization. However, to strengthen the validity of the findings, such an experiment should be confirmed by different well-experienced invasive cardiologists of different institutions. 

While the sample size was sufficient for the primary measurements, certain patient subgroups, such as those with chronic kidney disease who might uniquely benefit from dTRA, were not included. Furthermore, while most procedures utilized standard vascular sheaths, the potential benefits of slender sheaths, which may reduce vessel trauma, warrant further investigation in the context of dTRA. Our study was limited by its focus on a single patient population and did not differentiate between acute and elective cases, which may have distinct anatomical considerations for the dTRA. It is important to note that the study population consisted exclusively of Caucasians; therefore, these findings may not be generalizable to other racial or ethnic groups. Finally, the potential influence of occupational activity on vessel size, a factor that could impact dTRA outcomes, was not assessed in this study. 

## 5. Conclusions

This study underscores key anatomical considerations for dTRA. The distal RA, being inherently smaller than the proximal segment, necessitates careful pre-procedural assessment. Notably, both sex and body size, particularly BSA, emerged as independent predictors of distal RA diameter. This highlights the crucial role of pre-procedural DUS, especially in women and individuals with smaller BSA, to ensure adequate access and minimize potential complications. The positive correlation observed between distal RA diameter and palpable pulse strength suggests that meticulous palpation could serve as an initial, albeit less precise, estimate of distal RA size. While dTRA led to an immediate dilation of both distal and proximal RA segments, this effect persisted only in the distal segment at 60 days. A thorough understanding of distal RA anatomy is essential for optimizing patient selection and refining techniques for transradial procedures. Our findings support routine pre-procedural ultrasound, alongside clinical judgment, to optimize TRA site selection. 

## Figures and Tables

**Table 1:** Baseline patient clinical characteristics.

	Overall n = 348	Distal TRA n = 169	Conventional TRA n = 179	*p*-Value (Distal vs. Conventional)
Demographics				
Age, years; Avg (SD)	66.6 (10)	66.6 (9.4)	66.7 (10.5)	0.95
Female; N (%)	129 (37)	62 (36.7)	67 (37.4)	0.89
Height, m; M (IQR)	1.69 (1.61–1.75)	1.69 (1.61–1.75)	1.70 (1.61–1.76)	0.68
Weight, kg; M (IQR)	83.5 (72–96)	84 (72–97)	83 (72–95)	0.34
Body mass index, kg/m^2^; M (IQR)	29 (26–33)	29.8 (25.9–34.5)	28.4 (26.0–32.4)	0.15
Body surface area, m^2^; Avg (SD)	1.94 (0.22)	1.95 (0.23)	1.93 (0.22)	0.36
Comorbidities				
Hypertension; N (%)	277 (79.6)	137 (81.1)	140 (78.2)	0.51
Diabetes mellitus; N (%)	121 (34.9)	56 (33.1)	65 (36.3)	0.53
Dyslipidemia; N (%)	228 (65.5)	111 (65.7)	117 (65.4)	0.95
Smoking; N (%)	158 (45.4)	77 (45.6)	81 (45.2)	0.95
Obesity (BMI > 30); N (%)	154 (44.2)	81 (47.9)	73 (40.8)	0.18
COPD; N (%)	66 (19.0)	31 (18.3)	35 (19.5)	0.79
Peripheral artery disease; N (%)	34 (9.4)	18 (10.6)	16 (8.9)	0.59
Previous MI; N (%)	76 (21.8)	34 (20.1)	42 (23.5)	0.45
Previous stroke; N (%)	22 (6.3)	14 (8.3)	8 (4.5)	0.14
Previous CABG; N (%)	16 (6.3)	5 (2.9)	11 (6.1)	0.25
Previous PCI; N (%)	101 (29.0)	49 (29.0)	52 (29.0)	0.99
Indication for procedure				
Stable CAD; N (%)	202 (58)	103 (61)	99 (55.3)	0.29
Unstable angina; N (%)	62 (17.8)	29 (17.2)	33 (18.4)	0.76
NSTEMI; N (%)	44 (12.6)	20 (11.9)	24 (13.4)	0.66
Heart failure; N (%)	18 (5.2)	5 (2.9)	13 (7.3)	0.12
Ventricular arrhythmia; N (%)	10 (2.9)	5 (2.9)	5 (2.8)	0.82
Planned heart valve/aorta surgery; N (%)	12 (3.5)	7 (4.1)	5 (2.8)	0.69
Periprocedural oral medication				
Aspirin; N (%)	343 (98.6)	167 (98.8)	176 (98.3)	0.95
P2Y12 inhibitor; N (%)	232 (66.7)	117 (69.2)	115 (64.2)	0.32
DOAC (unstopped therapy); N (%)	1 (0.3)	0 (0)	1 (0.6)	0.98
Laboratory data				
Hemoglobin, g/dL; M (IQR)	14.1 (13.2–14.9)	14.0 (13.1–14.9)	14.1 (13.2–15.0)	0.49
Platelets, K/µL; M (IQR)	225 (187–274)	227 (189–274)	224 (184–272)	0.40
eGFR, mL/min; M (IQR)	84 (68–104)	83.8 (67.0–100.5)	84.7 (68–106)	0.72
LVEF, %; M (IQR)	55 (50–60)	55 (50–60)	55 (45–60)	0.37
Radial artery pulse strength (0—no pulse, 1—moderately, and 2—well palpable pulse)				
Distal radial artery, N (%)	10 (2.9), 229 (65.8), 109 (31.3)	3 (1.8), 111 (65.7), 55 (32.5)	7 (3.9), 118 (65.9), 54 (30.2)	0.49
Proximal radial artery, N (%)	3 (0.9), 130 (37.3), 215 (61.8)	1 (0.6), 57 (33.7), 111 (65.7)	2 (1.1), 73 (40.8), 104 (58.1)	0.37

Data presented are medians and interquartile range (IQRs), averages and standard deviations (SDs), or numbers (%). Continuous variables were compared using the unpaired *t* test or Mann–Whitney U test. Categorical variables were compared using chi-square analyses or the Fisher exact test. A = average; BMI = body mass index; CABG = coronary artery bypass grafting; CAD = coronary artery disease; COPD = chronic obstructive pulmonary disease; DOAC = direct oral anticoagulants; eGFR = estimated glomerular filtration rate; IQR = interquartile range; LVEF = left ventricular ejection fraction; M = median; MI = myocardial infarction; N = number; NSTEMI = non ST-segment-elevation myocardial infarction; PCI = percutaneous coronary intervention; P2Y12 inhibitor = platelet P2Y12 receptor inhibitor; SD = standard deviation; TRA = transradial approach.

**Table 2:** Procedural and angiographic data.

	Overall n = 348	Distal TRA n = 169	Conventional TRA, n = 179	*p*-Value (Distal vs. Conventional)
Prior ipsilateral TRA; N (%)	85 (24.4)	40 (23.7)	45 (25.1)	0.75
Right-sided TRA; N (%)	215 (61.8)	106 (62.7)	109 (60.9)	0.73
Access performance time, s; M (IQR)	90 (67–160)	115 (80–230)	80 (55–120)	<0.001
Arterial sheath size; N (%)				
5-Fr	144 (41.4)	87 (51.5)	57 (31.8)	<0.001 <0.001
Including 6-Fr GS	8 (2.3)	8 (4.7)	0	
6-F	196 (56.3)	74 (43.8)	122 (68.2)	
Procedure; N (%)				
CAG only	213 (61.2)	105 (62.1)	108 (60.3)	0.73
PCI only	51 (14.7)	22 (13)	29 (16.2)	0.40
CAG and PCI	84 (24.1)	42 (24.9)	42 (23.5)	0.76
Extent of CAD; N (%)				
No changes	80 (23.0)	39 (23.1)	41 (22.9)	0.49
Nonobstructive CAD	40 (11.5)	24 (14.2)	16 (8.9)	
1-VD	70 (20.1)	31 (18.3)	39 (21.8)	
2-VD	70 (20.1)	33 (19.5)	37 (20.7)	
3-VD or LMD ± any vessel	88 (25.3)	42 (24.9)	46 (25.7)	
Coronary artery treated; N (%)				
Left anterior descending artery	61 (17.5)	33 (19.5)	28 (15.6)	0.56
Left circumflex artery	29 (8.3)	12 (7.1)	17 (9.5)	
Right coronary artery	42 (12.1)	19 (11.2)	23 (12.8)	
Left main coronary artery	2 (0.6)	0	2 (1.1)	
Unfractionated heparin; N (%)				
2500 IU	212 (60.9)	105 (62.1)	107 (59.8)	0.65
≥5000 IU	136 (39.1)	64 (37.9)	72 (40.2)	0.65
Fluoroscopy time, min; M (IQR)	4.02 (1.98–7.99)	3.76 (1.90–8.31)	4.07 (2.00–7.74)	0.76
Fluoroscopy effective dose, mGy; M (IQR)	231 (107–446)	235 (116–478)	214 (99–429)	0.30
Total procedure time, min; M (IQR)	16.8 (10–32.4)	17.0 (10.3–34.0)	16.5 (9.7–30.5)	0.26

Data presented are medians (IQRs) or numbers (%). Continuous variables were compared using the unpaired *t* test or Mann–Whitney U test. Categorical variables were compared using chi-square analyses or the Fisher exact test. CAD = coronary artery disease; CAG = coronary angiography; Fr = French; GS = Glidesheath Slender^®^ introducer (Terumo); IQR = interquartile range; IU = international unit; LMD = left main coronary artery disease; M = median; Min—minute; N = number; PCI = percutaneous coronary intervention; Sec = second; TRA = transradial approach; VD = coronary vessel disease.

**Table 3:** Gender-related initial measurements of arteries diameter and blood flow.

	Overall n = 400	Male n = 241	Female n = 159	*p*-Value (Male vs. Female)
Artery lumen diameter, mm, Avg (SD)				
Distal RA	2.21 (0.33)	2.33 (0.31)	2.04 (0.27)	<0.0001
Proximal RA	2.47 (0.33)	2.60 (0.31)	2.28 (0.26)	<0.0001
Ulnar	2.12 (0.36)	2.22 (0.35)	1.97 (0.32)	<0.0001
Peak velocity flow, m/s, Avg (SD)				
Distal RA	0.58 (0.16)	0.58 (0.16)	0.58 (0.16)	0.87
Proximal RA	0.61 (0.15)	0.61 (0.15)	0.63 (0.16)	0.15
Ulnar	0.57 (0.15)	0.57 (0.16)	0.58 (0.15)	0.13

Data presented are average (SD). Avg = average; mm = millimeter; m/s = meter per second; RA = radial artery; SD = standard deviation.

**Table 4:** Univariate predictors of the lumen diameter of the radial artery.

	Distal Radial Artery			Proximal Radial Artery		
	Beta Coefficient (β)	95% CI	*p*-Value	Beta Coefficient (β)	95% CI	*p*-Value
Age	−0.001	−0.005–0.002	0.35	−0.002	−0.005–0.001	0.21
Height	1.19	0.874–1.514	<0.0001	1.38	1.069–1.698	<0.0001
Weight	0.007	0.006–0.009	<0.0001	0.008	0.006–0.009	<0.0001
Body mass index	0.013	0.008–0.020	<0.0001	0.013	0.007–0.019	<0.0001
Body surface area	0.62	0.490–0.757	<0.0001	0.67	0.542–0.807	<0.0001
Hypertension	0.066	−0.013–0.145	0.10	0.038	−0.042–0.118	0.35
Diabetes	−0.009	−0.079–0.059	0.79	−0.015	−0.084–0.055	0.67

CI = confidence interval.

**Table 5:** Pre- and post-procedural radial artery dimension and blood velocity flow.

	Distal TRA n = 169	Conventional TRA n = 179	*p*-Value
Distal RA diameter, mm; Avg (SD)			
Before procedure	2.24 (0.31)	2.23 (0.35)	0.57
1 day after procedure	2.55 (0.30)	2.25 (0.36)	<0.005
60 days after procedure	2.34 (0.32)	2.27 (0.34)	0.03
Proximal RA diameter, mm; Avg (SD)			
Before procedure	2.52 (0.33)	2.49 (0.33)	0.43
1 day after procedure	2.75 (0.32)	2.81 (0.33)	0.07
60 days after procedure	2.53 (0.35)	2.50 (0.32)	0.54
Distal RA peak velocity flow, m/s; Avg (SD)			
Before procedure	0.59 (0.16)	0.57 (0.16)	0.28
1 day after procedure	0.58 (0.19)	0.62 (0.18)	0.01
60 days after procedure	0.63 (0.17)	0.58 (0.16)	<0.01
Proximal RA peak velocity flow, m/s; Avg (SD)			
Before procedure	0.63 (0.15)	0.60 (0.14)	0.22
1 day after procedure	0.60 (0.17)	0.53 (0.18)	<0.005
60 days after procedure	0.67 (0.17)	0.66 (0.18)	0.24
Vascular sheath			
Sheath outer diameter, mm; Avg (SD)	2.44 (0.16)	2.51 (0.15)	<0.001
Punctured artery/sheath ratio, mm; Avg (SD)	0.92 (0.11)	0.99 (0.13)	<0.001

Data presented are average (SD). Avg = average; mm = millimeter; m/s = meter per second; RA = radial artery; TRA = transradial approach; SD = standard deviation.

## Data Availability

The data underlying this article will be shared on reasonable request to the corresponding author.
